# Impact of venous closure systems on time to ambulation and discharge following AF ablation: a systematic review and meta-analytic review

**DOI:** 10.1186/s43044-025-00685-5

**Published:** 2025-09-15

**Authors:** Muhammad Ashir Shafique, Hira Mustafa Nagra, Aisha Farooq, Sajal Ahmad, Syed Muhammad Fahad Gardezi, Usman Faisal, Muhammad Hamza Anees, Hafiz Muhammad Haris, Iman Moradi, Mathew Fredericks, Hassaan Dar, Behrooz Shojai Rahnama, Sher Ali Khan, Saif Khalid, Javed Iqbal, Janta Devi

**Affiliations:** 1 Jinnah Sindh Medical University, Karachi, Pakistan; 2 Shaikh Khalifa Bin Zayed Al Nahyan Medical and Dental College, Lahore, Pakistan; 3 Jiujiang Medical University, Jiujiang, China; 4 CMH Lahore Medical College and Institute of Dentistry, Lahore, Pakistan; 5 Sharif Medical and Dental College, Lahore, Pakistan; 6 King Edward Medical University, Lahore, Pakistan; 7 St. Georges University, St. Georges, Grenada; 8 Jinnah Medical & Dental College, Karachi, Pakistan; 9 Royal College of Surgeons in Ireland, Dublin, Ireland; 10 University of Chicago, Chicago, United States; 11 Liaquat University of Medical and Health Science, Jamshoro, Pakistan

**Keywords:** Atrial fibrillation, Pulmonary vein isolation, Venous closure system, Perclose, Manual compression, Hemostasis, Time to ambulation, Time to discharge, Vascular access complications, Meta-analysis

## Abstract

**Background:**

Atrial fibrillation (AF) is the most common sustained cardiac rhythm disorder, significantly impacting global health and healthcare costs. Pulmonary vein isolation (PVI) is the preferred method for catheter-based AF ablation, reducing arrhythmia recurrence. However, vascular access complications remain a concern. This systematic review and meta-analysis aimed to compare the efficacy and safety of venous closure systems (VCSs), like Perclose™ ProGlide™, with traditional manual compression (MC) techniques, focusing on time to hemostasis (TTH), time to ambulation in hours (TTA), time to discharge (TTD), and complication rates.

**Method:**

A comprehensive search was conducted in PubMed, Medline, Scopus, and Embase, adhering to PRISMA guidelines. Five studies met the inclusion criteria, comprising randomized controlled trials (RCTs) and observational studies. Data were analyzed using OpenMeta, applying a random-effects model to calculate standardized mean differences (SMDs) and odds ratios (ORs). Heterogeneity was assessed using the *I*^2^ statistic, and funnel plots evaluated publication bias.

**Result:**

The meta-analysis included 5 studies with a total of 240 patients. VCSs significantly reduced TTA (SMD − 2.029, 95% CI − 3.097 to − 0.962, *p* = 0.001) and TTD (SMD − 2.081, 95% CI − 3.870 to − 0.292, *p* = 0.023) compared to MC, but showed no significant reduction in TTH (SMD − 1.109, 95% CI − 2.524 to 0.307, *p* = 0.125). No significant differences were observed in bleeding complications (OR 1.35, 95% CI 0.413 to 4.125, *p* = 0.604) or hematoma rates (OR 4.665, 95% CI 0.768 to 28.345, *p* = 0.094).

**Conclusion:**

VCSs demonstrated faster ambulation and discharge times compared to MC techniques, suggesting potential benefits in improving patient flow and satisfaction. However, the slight increase in hematoma risk warrants further investigation. These findings could guide clinical decision-making in vascular access management post-AF ablation.

**Supplementary Information:**

The online version contains supplementary material available at 10.1186/s43044-025-00685-5.

## Background

Atrial fibrillation (AF), the most prevalent persistent cardiac rhythm disorder, exerts a substantial influence on global morbidity, mortality, and healthcare expenditure [1–3]. Pulmonary vein isolation (PVI) has become the primary approach in catheter-based ablation therapy for AF, effectively diminishing arrhythmia recurrence and enhancing patient outcomes. The increasing demand for PVI procedures has been accompanied by the evolution of sophisticated ablation technologies, including cryoballoon-based PVI and pulsed field ablation (PFA) [4–7]. These innovative techniques have gained favor due to their reduced learning curves, lower complication rates, and the potential for same-day discharge (SDD) protocols, which aim to boost patient satisfaction and curtail healthcare costs [8–10].

Notwithstanding these improvements, complications related to vascular access remain a notable issue in catheter-based atrial fibrillation ablation. Pseudoaneurysm, arteriovenous fistula, and retroperitoneal hemorrhage are the most prevalent complications, often aggravated by the quantity and dimensions of sheaths employed [11–13]. Moreover, the rigorous periprocedural anticoagulation necessary to avert thromboembolism further heightens the risk of bleeding and hematoma formation, presenting obstacles to achieving swift and efficacious hemostasiss [14–16].

Historically, the conventional method for managing vascular access sites following procedures has been manual compression (MC), sometimes accompanied by a figure-of-eight suture [17, 18]. Although these techniques are efficacious, they often result in extended time to ambulation (TTA) and delayed discharge eligibility, which can impede the successful execution of SDD protocols. In recent years, innovative alternatives to MC have emerged in the form of venous closure systems (VCSs), such as the Perclose™ and ProGlide™ [19, 20]. These novel devices are designed to decrease TTA, time to hemostasis (TTH), and time to discharge eligibility (TTDe), while simultaneously improving patient comfort and safety.

In light of the persistent issues surrounding vascular access management in AF ablation, there is an urgent requirement to assess the effectiveness and safety of newer VCS technologies in comparison with conventional approaches. This comprehensive review and statistical analysis aim to offer a thorough evaluation of the current evidence regarding the utilization of VCSs such as Perclose™ versus traditional manual compression with figure-of-eight sutures. The primary goals are to contrast these interventions in terms of TTA, TTH, TTDe, the frequency of vascular access complications, and overall patient contentment. Through the consolidation of existing data, this investigation endeavors to guide clinical practice and inform decision-making in the realm of vascular access management following AF ablation, ultimately contributing to enhanced patient outcomes and more efficient utilization of healthcare resources.

## Methods

Adhering to the Cochrane Handbook guidelines and PRISMA (Preferred Reporting Items for Systematic Reviews and Meta-Analyses) principles [21], a systematic review and meta-analysis were carried out in accordance with the established protocol.

### Search strategy

A thorough search was carried out across multiple databases, including PubMed, Medline, Scopus, and Embase, to gather all relevant data up until January 03, 2025. The search strategy incorporated Mesh terms and common keywords such as “Perclose ProGlide,” “Figure-of-Eight Suture,” “Venous Closure System,” and related terms. The search was conducted in English, and additional resources were utilized to identify pertinent studies. Two researchers independently created the search strategy, ensuring adherence to the criteria, and any inconsistencies were settled with the aid of a third reviewer.

### Eligibility criteria

Studies were considered if they evaluated venous closure systems (excluding Perclose only) in comparison with conventional techniques for large-bore venous access hemostasis in grown-up patients. The studies that were eligible were randomized controlled trials (RCTs), quasi-RCTs, cohort studies, and observational studies. The studies that were not included were case reports, review articles, letters, narrative reviews, commentaries, and those published in languages other than English.

### Data extraction

Two evaluators separately obtained information from the suitable studies. In case of discrepancies, a third arbitrator was consulted. The details collected comprised of the author’s name, year of publication, location of the study, number of participants, features of the patients such as age and body mass index, duration of follow-up, particulars of the intervention and the end results, which included time to hemostasis (TTH), time to ambulation (TTA), time to discharge (TTD), bleeding, and hematoma rates. The information was scrutinized meticulously for any duplication or inconsistencies prior to the analysis.

### Data analysis

The OpenMeta software was employed for data analysis. Given the anticipated variation across studies, a random-effects model was implemented to pool the results. Standardized mean differences (SMDs) were computed for continuous outcomes, such as TTH, TTA, and TTD, while odds ratios (ORs) were applied to dichotomous outcomes, such as bleeding and hematoma, along with their respective 95% confidence intervals. The *I*^2^ statistic was utilized to assess heterogeneity, and sensitivity analyses were conducted to pinpoint potential sources of heterogeneity. To evaluate publication bias, funnel plots were created.

### Quality assessment and risk of bias

The Cochrane Risk of Bias tool was employed to evaluate the quality of the RCTs by examining factors such as random sequence generation, allocation concealment, blinding, and outcome reporting. Each RCT was assigned a rating of low, high, or unclear risk of bias (Supp Fig. 1 and 2). The Newcastle–Ottawa Scale (NOS) was utilized to assess the quality of cohort and observational studies by considering selection, comparability, and outcome assessment criteria, with scores ranging from zero to nine. In case of any discrepancies in quality assessment, a third reviewer was consulted to ensure consistency and accuracy.

## Results

### Literature search

**Fig. 1:**
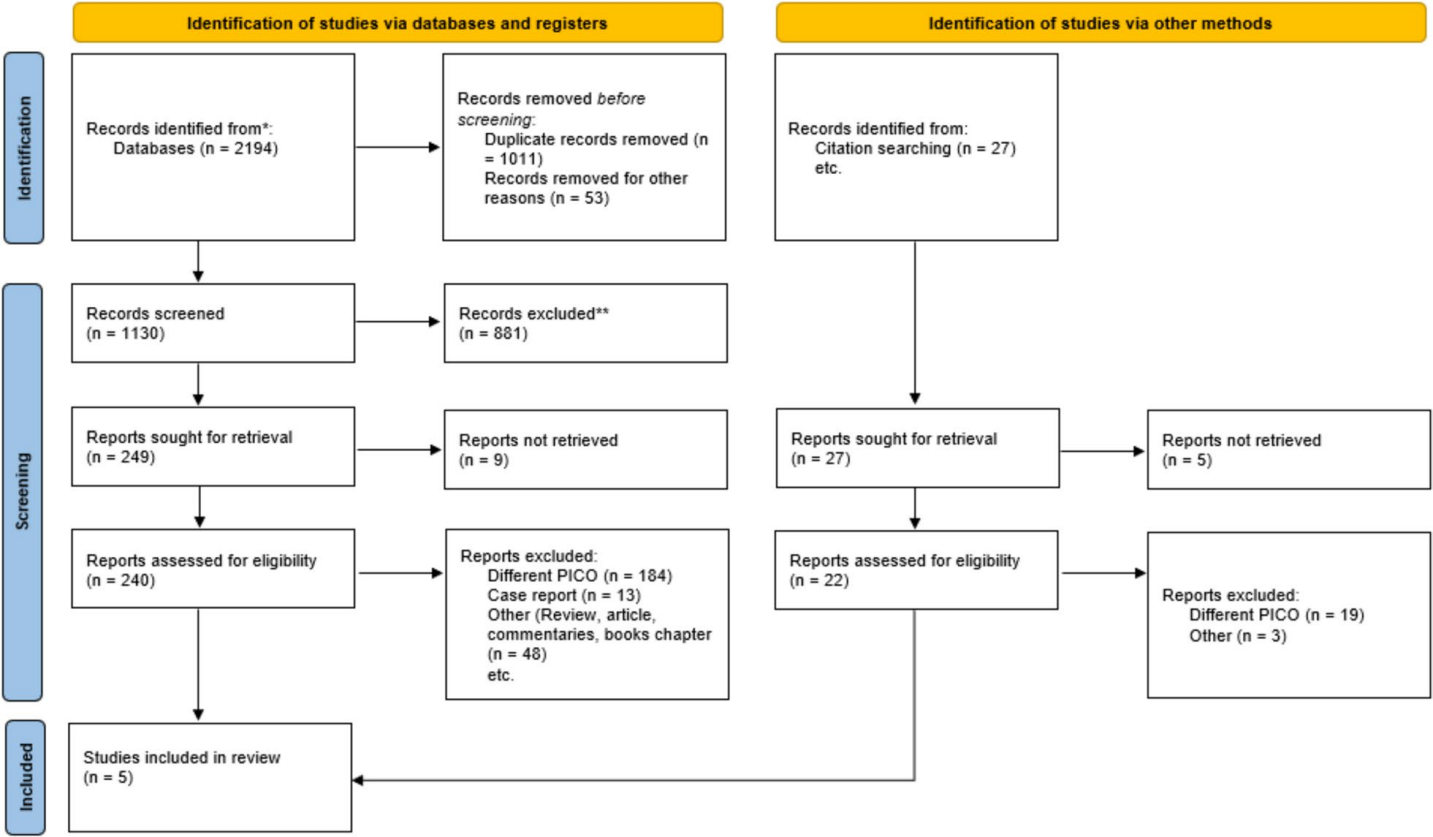
PRISMA flow chart for the literature search

A comprehensive search was conducted across multiple databases, yielding a total of 2194 records. After removing 1011 duplicate records and 53 records for other reasons, 1130 records remained for screening. Out of these, 881 records were excluded based on title and abstract screening, leaving 240 reports for full-text assessment. Of these, 184 reports were excluded due to a different PICO (Population, Intervention, Comparison, Outcome) framework, 13 were case reports, and 48 were other types of publications such as reviews, articles, commentaries, and book chapters. Ultimately, five studies met the inclusion criteria. Among these, two were randomized controlled trials and three were cohort studies (Fig. 1).

### Baseline characteristics

The baseline characteristics of the included studies varied across different countries and populations. Ali et al. (2024, USA) compared the PPG suture-mediated closure system with the figure-of-eight suture in 47 and 53 patients, respectively, with mean ages around 69.5 years, and reported higher male representation and BMI in the intervention group. Comorbidities included diabetes mellitus (DM), hypertension (HTN), chronic kidney disease (CKD), end-stage liver disease, and stroke. Fabbricatore et al. (2023, Italy) examined 50 patients using the PPG system, with a median age of 64 years and notable comorbidities like DM, HTN, and smoking. Lodhi et al. (2023, USA) compared 20 patients each in the PPG and figure-of-eight groups, with mean ages over 81 years and similar male distributions, BMI, and comorbidities (DM, HTN, smoking). Castro-Urda et al. (2023, Spain) analyzed 50 patients per group with median ages around 65 years, slightly higher male representation in the control group, and similar comorbidities. Lastly, Tilz et al. (2024, USA) studied 63 and 62 patients in the PPG and figure-of-eight groups, respectively, with median ages around 64–68 years, and comorbidities including DM, HTN, smoking, and CKD. Across all studies, the intervention groups generally showed comparable or slightly higher baseline comorbidities and BMI compared to the control groups (Table 1).

**Table 1:** Baseline characteristics of included studies

Author	Country	Type		Sample size		Age		Gender (Male)		BMI		Comorbs	
		Intervention	Control	Intervention	Control	Intervention	Control	Intervention	Control	Intervention	Control	Intervention	Control
Ali et al., 2024	USA	PPG suture-mediated closure system	figure of 8 suture	47	53	69.5 ± 10.3	69.6 ± 11.7	27	36	31.8 ± 7.1	30.3 ± 6.6	DM (10), HTN (27), CKD (3), End-stage Liver disease (2), Stroke (6)	DM (11), HTN (31), CKD (6), End-stage Liver disease (0), Stroke (7)
Fabbricatore et al., 2023	Italy	PPG suture-mediated closure system		50		64 (28–80)		38		26.3 (21–42)		DM (11), HTN (20), Smokers (4)	
Lodhi et al., 2023	USA	PPG suture-mediated closure system	Figure of 8	20	20	81.3 (7.9)	83.3 (8.3)	13	12	25.5 (6.1)	27.3 (5.5)	DM (5), HTN (19), Smokers (6)	DM (5), HTN (19), Smokers (6)
Castro-Urda et al., 2023	Spain	PPG suture-mediated closure system	Figure of 8	50	50	65 (58.75–69,75)	63 (54.25–68)	32	37	26.73 (24.57–31.19)	26.88 (23.90–29.98)	DM (11), HTN (25), Smokers (42)	DM (4), HTN (19), Smokers (24)
Tilz et al., 2024	USA	PPG suture-mediated closure system	Figure of 8	63	62	64.0 (56.0, 74.0)	68.5 (60.8, 75.3)	38	42	29.4 ± 5.6	27.5 ± 4.7	DM (5), HTN (40), Smoker (6), CKD (11)	DM (7), HTN (36), Smoker (2), CKD (8)

### Primary outcomes

**Fig. 2:**
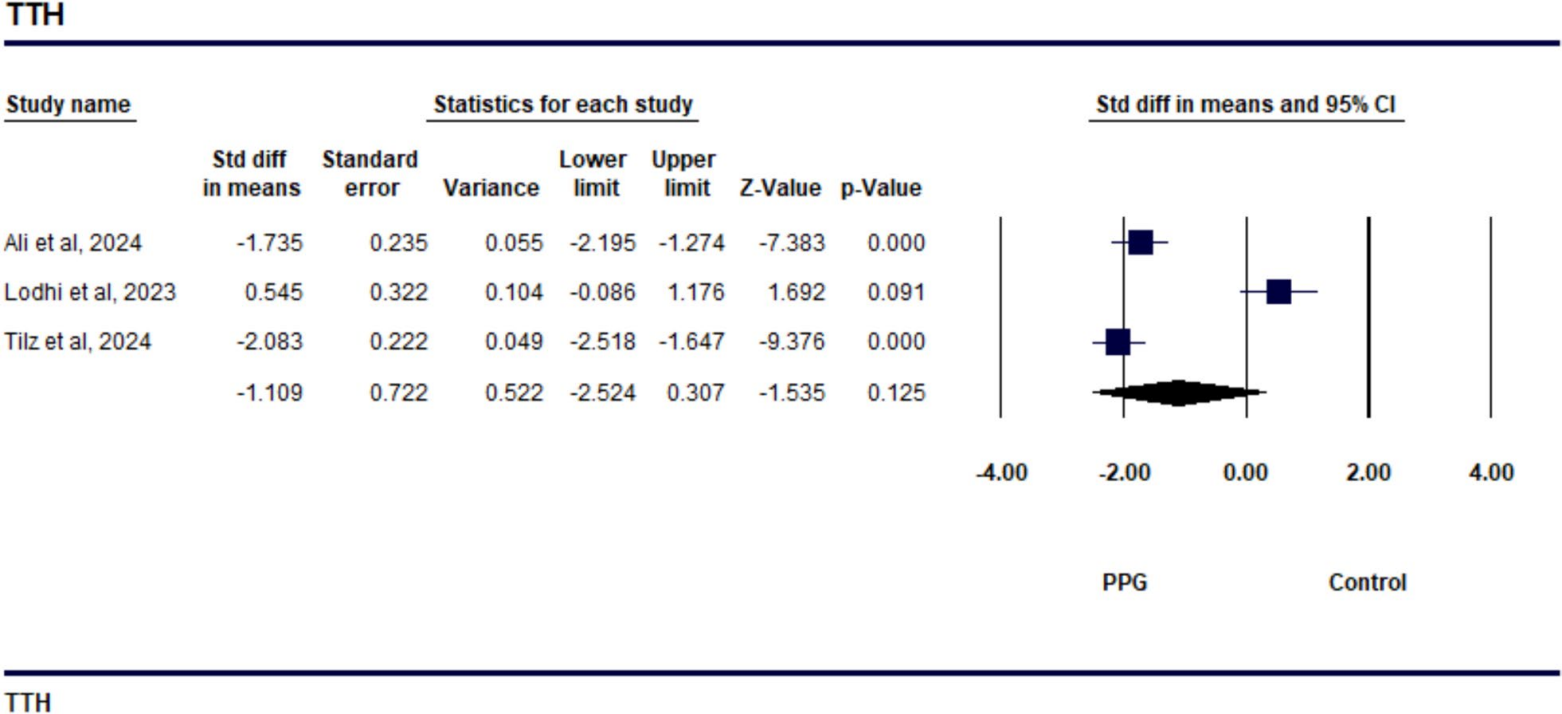
Forest plot comparing Time to Hemostasis (TTH) between venous closure systems (VCSs) and traditional methods

**Fig. 3:**
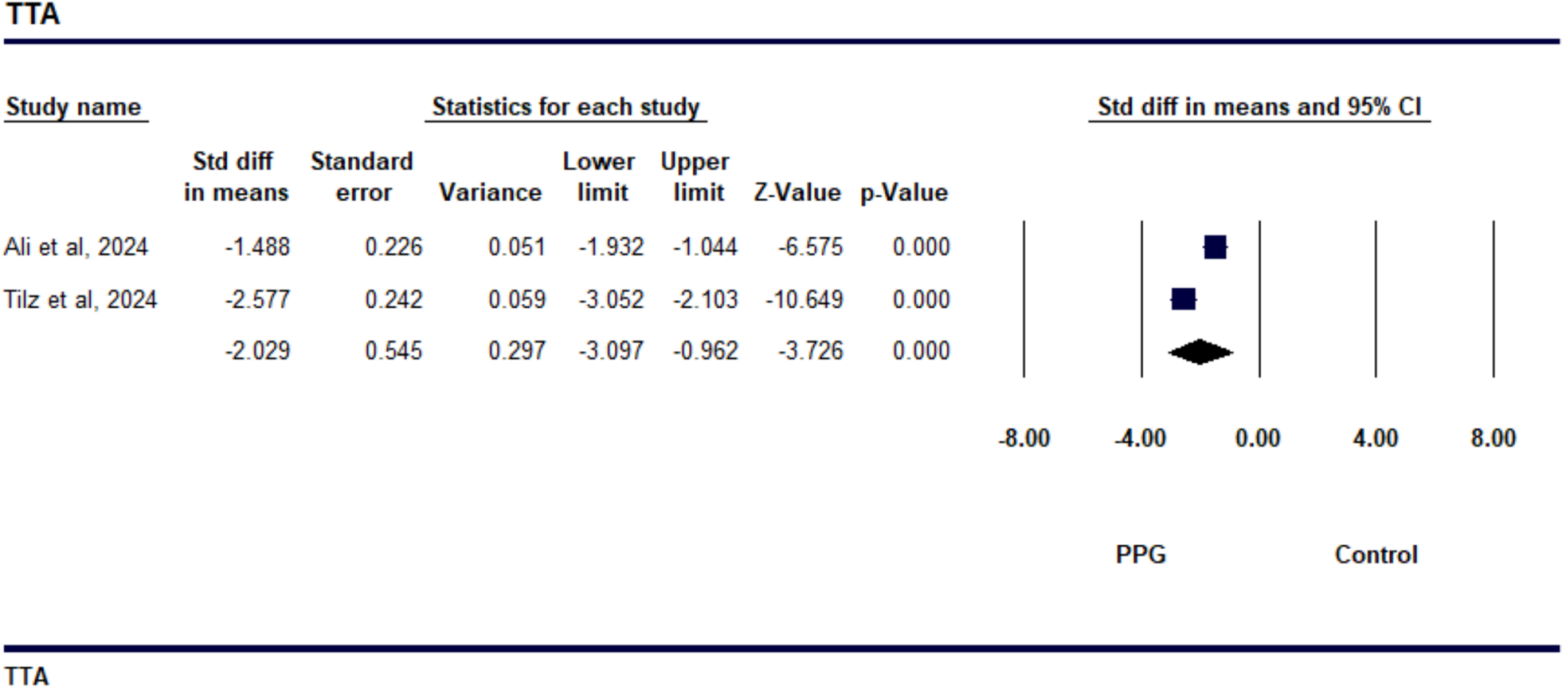
Forest plot illustrating the effect of venous closure systems (VCSs) versus traditional methods on time to ambulation (TTA)

**Fig. 4:**
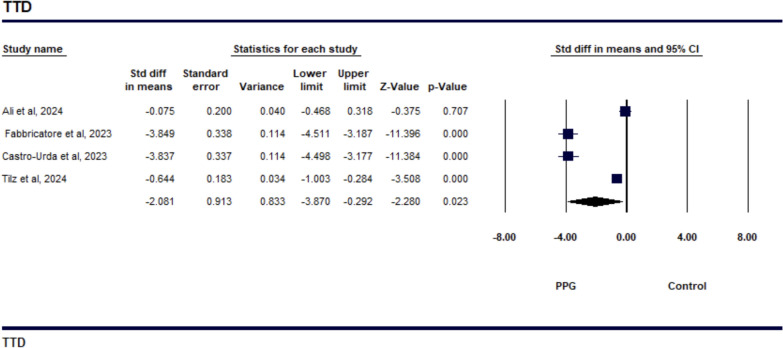
Forest plot showing the comparison of time to discharge (TTD) between VCSs and traditional methods following PVI

The study on TTH demonstrated a SMD of − 1.109 (95% CI − 2.524 to 0.307), indicating a reduction in TTH with the PPG suture-mediated closure compared to the figure-of-eight suture technique, as shown in Fig. 2. However, this result was not statistically significant (*p* = 0.125). The level of heterogeneity among the studies was high, with an *I*^2^ value of 95.8% (*p* = 0.001) (Supp Fig. 3), which suggests significant variability in the effect sizes across the studies. The sensitivity analysis revealed that the study by Lodhi et al. was a significant contributor to the observed heterogeneity. The studies reporting TTA showed a notable decrease in time when using the PPG suture-mediated closure compared to the figure-of-eight suture technique, with a SMD of − 2.029 (95% CI − 3.097 to − 0.962, *p* = 0.001), as depicted in Fig. 3. However, the analysis also revealed high heterogeneity among the studies, with an I^2^ value of 90.745% (*p* = 0.001), indicating considerable variability in the effect sizes across the studies included (as shown in Supp Fig. 4). The study comparing TTD showed that the PPG was more effective in reducing discharge time than the Standard suture technique, with a SMD of − 2.081 (95% CI − 3.870 to − 0.292, *p* = 0.023). This is illustrated in Fig. 4, indicating that patients using the Perclose technique were discharged significantly earlier. However, there was a high level of heterogeneity among the studies, with an *I*^2^ value of 96.164% (*p* = 0.001), which suggests considerable variability in the effect sizes (see Supp Fig. 5). All of the studies included in the analysis reported a greater reduction in TTD for Perclose. The sensitivity analysis revealed that the studies by Ali et al. and Tilz et al. contributed to the observed heterogeneity..

### Secondary outcomes

**Fig. 5:**
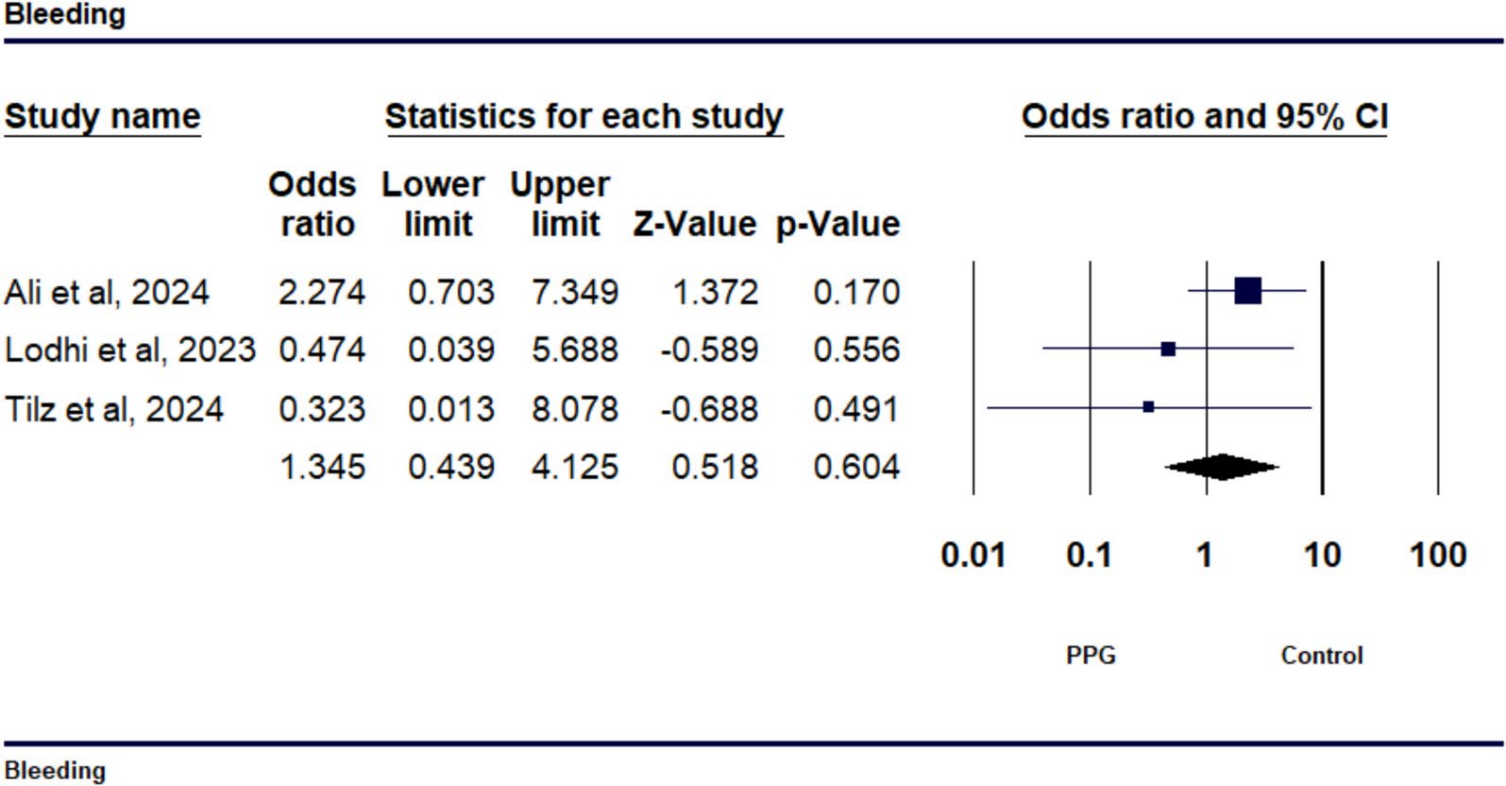
Forest plot comparing the incidence of bleeding between venous closure systems (VCSs) and traditional methods

**Fig. 6:**
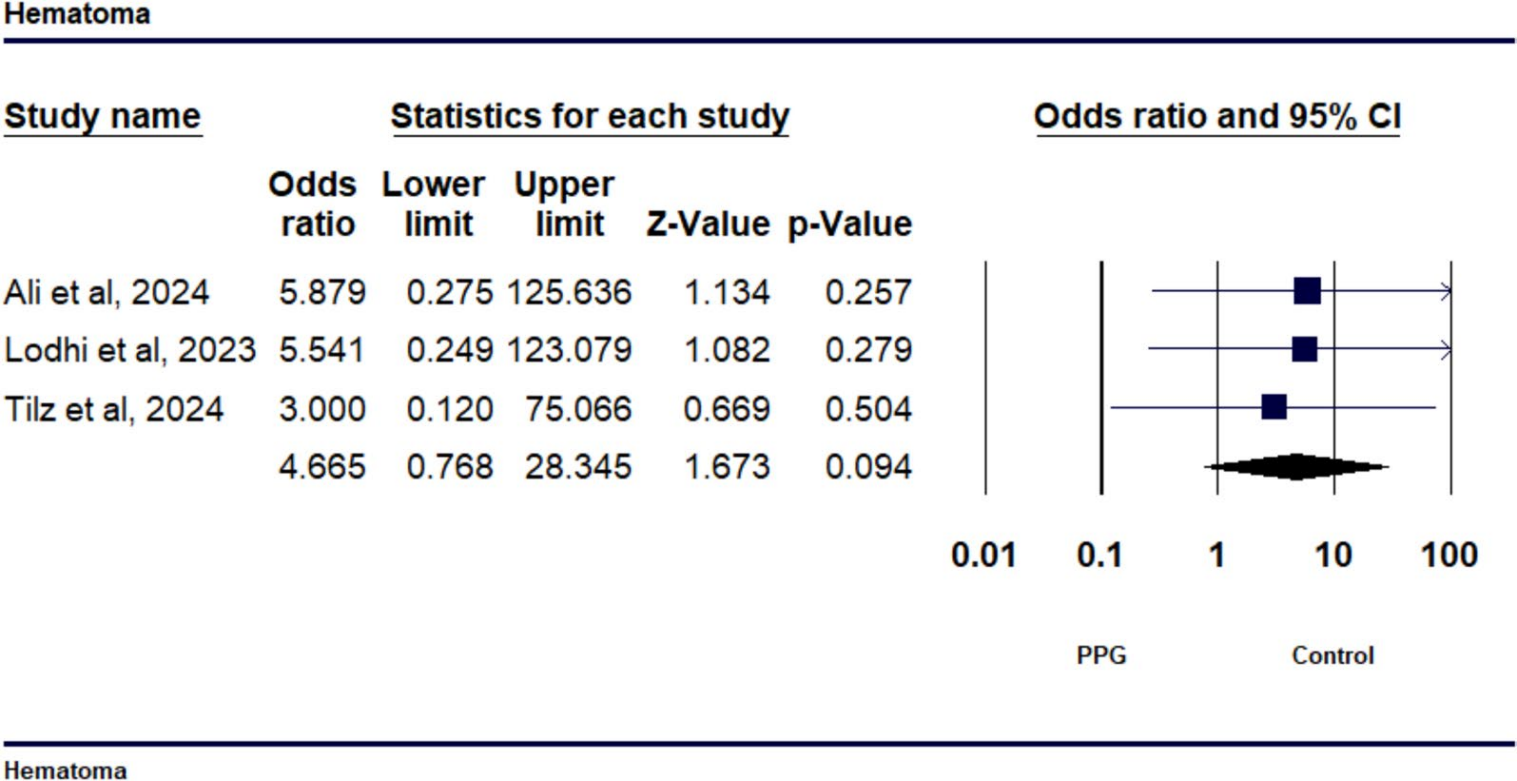
Forest plot of Hematoma outcomes following PVI: Venous closure systems (VCSs) versus traditional methods

The study’s findings regarding bleeding outcomes revealed an odds ratio (OR) of 1.35 (95% CI 0.4125 to 4.125, *p* = 0.604) when comparing the PPG suture-mediated closure to the figure-of-eight suture technique, as depicted in Fig. 5. This suggests that there is no statistically significant difference in the risk of bleeding between the two techniques. The analysis also showed low heterogeneity among the studies, with an I^2^ value of 8.227% (*p* = 0.336), indicating a consistent effect size across the studies (see Supp Fig. 6). The examination of hematoma results demonstrated an OR of 4.665 (95% CI 0.768 to 28.345, *p* = 0.094) when comparing the PPG suture-mediated closure technique to the figure-of-eight suture method, as depicted in Fig. 6. The finding is not statistically significant. The inconsistency among the studies was minimal, with an I^2^ value of 0.000% (*p* = 0.948), indicating consistent effect sizes across the studies (see supp Fig. 7).

## Discussion

Following pulmonary vein isolation (PVI) in atrial fibrillation (AF) ablation procedures, the handling of vascular access sites remains a crucial element of patient care, with direct implications for recovery duration, complication frequency, and overall healthcare resource consumption. The growing prevalence of catheter-based ablation, owing to its effectiveness in reducing AF recurrence, has heightened the demand for efficient and safe hemostasis techniques. While manual compression (MC), with or without a figure-of-eight suture, has long been the conventional approach, it often results in extended time to ambulation (TTA) and delayed discharge eligibility, potentially hindering same-day discharge (SDD) protocols. The introduction of venous closure systems (VCS) such as Perclose™ ProGlide™ and ProStyle™ offers a promising alternative, potentially reducing hemostasis times and enhancing patient satisfaction. This meta-analysis is essential as it consolidates existing evidence to assess the efficacy and safety of these innovative VCS technologies in comparison to traditional methods. By providing a thorough evaluation, this study aims to address gaps in current literature, guide clinical decision-making, and ultimately improve patient outcomes and healthcare efficiency.

The systematic review indicated that vascular closure devices, particularly the Perclose™ ProGlide™ and ProStyle™ systems, show a tendency toward shortened periods for achieving hemostasis, walking, and hospital discharge readiness when compared to conventional manual compression techniques using figure-of-eight sutures. Although the decrease in time to hemostasis was not statistically significant, there were notable reductions in time to ambulation and discharge eligibility. This suggests that patients treated with VCS may experience a more rapid return to normal activities and earlier discharge from healthcare facilities. Furthermore, the occurrence of bleeding complications was not significantly different between the two methods, though a non-significant trend toward increased hematoma risk was observed with the Perclose™ approach.

The meta-analysis revealed substantial heterogeneity in outcomes, likely due to factors such as the diversity in study protocols, particularly the quantity and dimensions of sheaths used during PVI procedures. This variability can affect the comparative effectiveness of VCS and conventional methods. Larger sheaths and multiple venous access points increase complication risks, necessitating more robust hemostatic approaches and impacting closure system performance. Additionally, variations in patient characteristics, such as age, BMI, and existing health conditions, influence the efficacy and safety of interventions. For instance, individuals with higher BMI or peripheral vascular disease may respond differently to VCS compared to MC due to differences in vascular anatomy and tissue quality, contributing to inconsistent effect sizes. Previous studies have indicated that individuals with higher BMI have a similar or increased risk of vascular complications compared to those with lower BMI [22, 23]. Notably, despite the VCS group having a significantly higher BMI, Tilz et al.[17] showed a trend toward fewer minor vascular access-related complications, supporting the safety of this approach. Procedural factors, such as operator experience and proficiency with VCS technologies, also influence outcomes. Centers with extensive experience using Perclose™ systems might report superior results and lower complication rates compared to less experienced settings. This operator-dependent variability introduces significant heterogeneity, as the learning curve associated with new technologies affects procedural success and complication rates.

The variation in anticoagulation protocols across studies could affect bleeding and hematoma rates [24, 25]. Differences in the strength and duration of anticoagulation therapy during and after ablation procedures may influence bleeding complications, regardless of the hemostasis technique used. These inconsistencies in anticoagulation management might obscure the true comparative efficacy of VCS versus MC. Additionally, varying follow-up periods among studies can contribute to heterogeneity in reported outcomes. Shorter follow-ups may capture immediate post-procedural complications but miss delayed events, while extended follow-ups provide a more comprehensive evaluation of hemostasis methods’ effectiveness and safety. These discrepancies in follow-up durations can lead to inconsistent reporting of bleeding and hematoma rates, complicating the interpretation of aggregated results.

The ability to conduct pulmonary vein isolation (PVI) and other advanced procedures in a day care setting is gaining attention within the electrophysiology (EP) field. Emerging evidence suggests that same-day discharge is feasible across various ablation techniques [26, 27]. Additionally, current findings indicate potential cost savings due to reduced hospital expenses, though there is limited prospective controlled data on this aspect [28, 29]. Multiple investigations, including those conducted by Kowalski et al6., Creta et al10., and Chu17, have consistently shown substantial financial benefits associated with implementing same-day discharge protocols [30–32]. These outcomes are in line with research by Víctor Castro-Urda et al. [19]., which revealed annual hospital cost reductions ranging from $45,825 to $83,813 across American medical facilities. Notably, the Víctor Castro-Urda study identified an average cost reduction of $379.16 per patient utilizing this approach. Extrapolating from these figures, assuming an 84% same-day discharge rate for a group of 200 annual cases not requiring general anesthesia, this would result in yearly savings of $75,832.

Tilz et al. [17] reported that patients in the VCS group experienced greater satisfaction and showed a tendency toward increased comfort with the time spent lying on their backs compared to the F8 group. Furthermore, when considering a hypothetical reduction in bed rest time, both groups expressed higher satisfaction and comfort with the shorter duration. These findings align with earlier studies that assessed patient satisfaction and comfort following catheter ablation procedures using VCS-based hemostasis, which also showed significantly higher satisfaction scores in the VCS group compared to the F8 group [33].

The observation that VCS did not markedly decrease the time to achieve hemostasis compared to manual compression merits additional investigation. This result could be attributed to the initial time required to set up VCS devices, potentially negating any time-saving benefits in attaining hemostasis. Moreover, the slight, albeit statistically insignificant, increase in hematoma risk associated with Perclose™ techniques implies that while these systems may accelerate hemostasis, they might also induce mechanical stress at the vascular access site, potentially leading to localized bleeding complications. When considering the implementation of VCS in clinical settings, it is crucial to weigh the advantages of reduced ambulation and discharge times against the possible increased risk of hematoma formation. On the other hand, the notable reduction in time to ambulation and discharge eligibility with VCS usage demonstrates a clear benefit in improving patient flow and satisfaction. Quicker ambulation not only enhances patient comfort but also diminishes the risk of venous stasis and associated complications. Similarly, accelerated discharge processes can ease the strain on healthcare systems, enabling greater procedural efficiency and shorter hospital stays, which is particularly beneficial in resource-limited environments. The lack of significant difference in overall bleeding rates between VCS and MC suggests that both methods are comparably safe in terms of major bleeding complications. However, the trend toward higher hematoma incidence with VCS indicates a need for careful patient selection and technique optimization when employing these devices. Training and standardization of VCS deployment protocols may mitigate this risk, ensuring that the benefits of reduced ambulation and discharge times are realized without compromising patient safety.

This meta-analysis is subject to several limitations that must be acknowledged. Firstly, the limited number of studies included restricts the generalizability of the findings and may affect the robustness of the conclusions drawn. The small sample sizes and varying study designs contribute to the high heterogeneity observed, particularly in time-related outcomes. Secondly, the included studies exhibited differences in follow-up durations, which could influence the detection and reporting of complications such as bleeding and hematoma. Shorter follow-up periods may underestimate the true incidence of these events, while longer follow-ups provide a more comprehensive assessment. Additionally, the protocols employed across studies varied, including differences in sheath sizes, anticoagulation regimens, and operator experience with VCS technologies. These discrepancies introduce variability that complicates the interpretation of pooled results and limits the ability to draw definitive conclusions.

Despite these limitations, this meta-analysis possesses several strengths that enhance its contribution to the existing body of literature. Notably, it represents the first comprehensive synthesis of evidence comparing venous closure systems to traditional manual compression methods in the context of AF ablation procedures. By aggregating data from multiple studies, this analysis provides a more robust evaluation of the relative efficacy and safety of VCS technologies, offering valuable insights that individual studies alone may not reveal. Furthermore, the systematic approach adhered to established guidelines, including the Cochrane Handbook and PRISMA principles, ensuring methodological rigor and minimizing bias. This study also identifies critical gaps in the literature, such as the need for standardized protocols and longer follow-up periods, thereby guiding future research endeavors aimed at optimizing vascular access management in AF ablation.

Looking ahead, future research should focus on conducting large-scale, multicenter randomized controlled trials with standardized protocols to further elucidate the comparative effectiveness of venous closure systems versus manual compression. Such studies should aim to harmonize variables like sheath size, anticoagulation strategies, and operator training to reduce heterogeneity and enhance the reliability of outcomes. Additionally, investigating patient-specific factors, such as vascular anatomy and comorbid conditions, could help identify subgroups that may benefit most from VCS technologies. Longitudinal studies with extended follow-up periods are also necessary to capture late-onset complications and assess the long-term safety and efficacy of these devices. Moreover, economic evaluations examining the cost-effectiveness of VCS compared to traditional methods would provide essential information for healthcare decision-makers considering the adoption of these technologies in clinical practice. Integrating patient-reported outcomes and satisfaction measures into future studies could further validate the benefits of VCS, ensuring that advancements in hemostasis methods align with patient-centered care objectives. Ultimately, continued innovation and rigorous evaluation will be pivotal in optimizing vascular access management strategies, enhancing patient outcomes, and ensuring the sustainable use of healthcare resources in the evolving landscape of AF ablation therapy.

## Conclusion

This systematic review and meta-analysis critically evaluate the effectiveness and safety of VCSs like Perclose™ ProGlide™ and ProStyle™ compared to traditional MC techniques in managing vascular access sites following PVI for AF ablation. The findings suggest that VCSs significantly reduce the time to ambulation and discharge eligibility, which could potentially enhance patient recovery and align with same-day discharge protocols. However, the reduction in time to hemostasis with VCSs was not statistically significant. Additionally, although bleeding rates were similar between VCSs and MC, there was a non-significant trend toward a higher risk of hematoma with VCSs. These results indicate that while VCSs offer benefits in terms of patient flow and satisfaction, attention must be given to the potential for increased hematoma risk. Future research should focus on large-scale, standardized trials to confirm these findings and address the identified limitations, including variations in study protocols and follow-up durations. Such research will be crucial for optimizing vascular access management strategies, improving patient outcomes, and ensuring the effective use of healthcare resources in AF ablation procedures.

## Supplementary Information

Additional file 1.

## Data Availability

No datasets were generated or analyzed during the current study.
