# Supplementary material for: Impact of venous closure systems on time to ambulation and discharge following AF ablation: a systematic review and meta-analytic review
Source: Egypt Heart J. 2025 Sep 15;77:88. doi: 10.1186/s43044-025-00685-5 (PMC12436246; doi:10.1186/s43044-025-00685-5)

**Supp Figure 1: Risk of Bias Assessment graph**


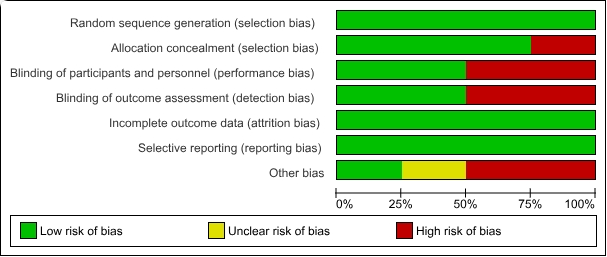


**Supp Figure 2: Risk of Bias Assessment summary**


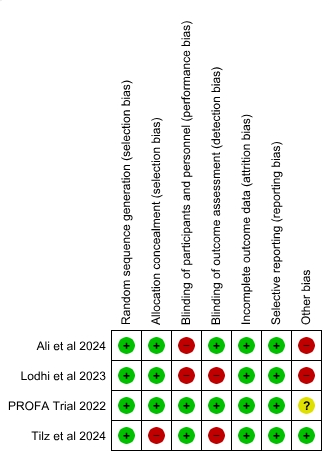


**Supplementary Figure 3: details of Forest plot comparing Time to Hemostasis (TTH) between venous closure systems (VCSs) and traditional methods**


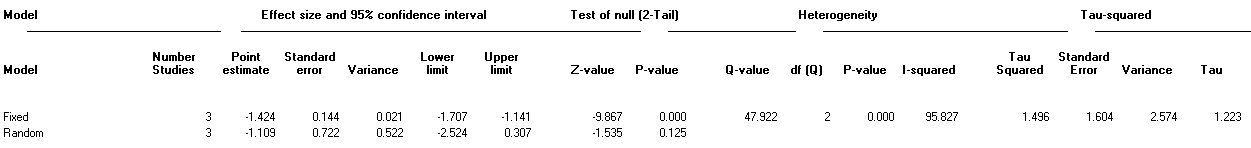


**Supplementary Figure 4: details of Forest plot comparing time to ambulation (TTA) between venous closure systems (VCSs) and traditional methods**


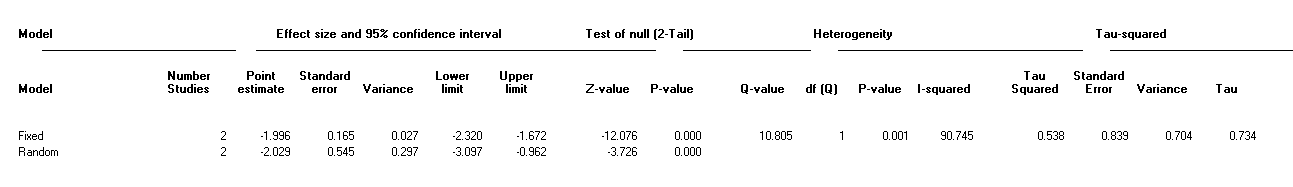


**Supplementary Figure 5: details of Forest plot comparing time to discharge (TTD) between venous closure systems (VCSs) and traditional methods**


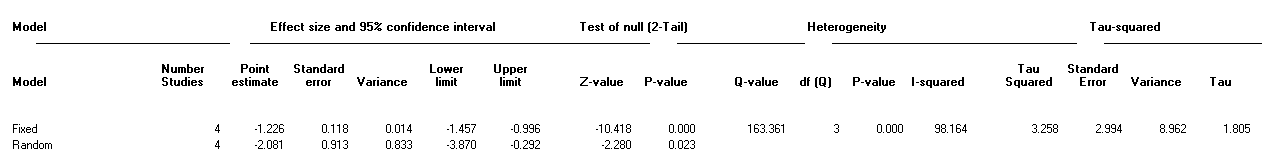


**Supplementary Figure 6: details of Forest plot comparing the incidence of bleeding between venous closure systems (VCSs) and traditional methods**


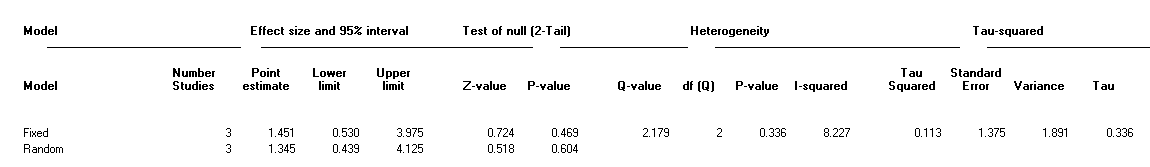


**Supplementary Figure 7: details of Forest plot comparing the incidence of hematoma between venous closure systems (VCSs) and traditional methods**


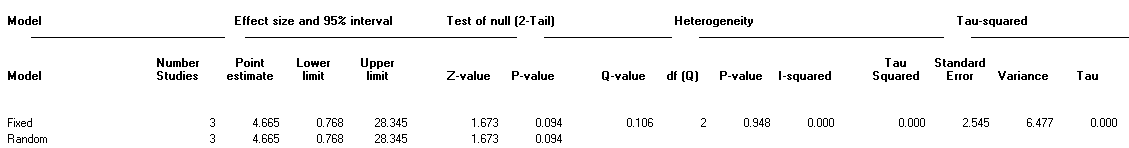

Supplement: Supplementary file 1 — Additional file 1. [file 43044_2025_685_MOESM1_ESM.docx]
